# Cosmesis and feasibility of transvaginal natural orifice Specimen extraction (NOSE) for large organ specimen: a prospective pilot study

**DOI:** 10.1186/s12894-022-01114-4

**Published:** 2022-10-29

**Authors:** Woo Yeon Hwang, Dong Hoon Suh, Sangchul Lee

**Affiliations:** 1 Department of Obstetrics and Gynecology, Seoul National University Bundang Hospital, Seongnam, Republic of Korea; 2 Department of Obstetrics and Gynecology, Seoul National University College of Medicine, Seoul, Republic of Korea; 3 Department of Urology, Seoul National University Bundang Hospital, Seongnam, Republic of Korea; 4 Department of Urology, Seoul National University Seoul National University College of Medicine, Seoul, Republic of Korea; 5 Present Address: Department of Obstetrics and Gynecology, Kyung Hee University Medical Center, Kyung Hee University College of Medicine, Seoul, Republic of Korea; 6 82 Gumi-ro, 173 Beon-gil, Bundang-gu, Seongnam-si, Gyeonggi-do, 13620 Republic of Korea

**Keywords:** Transvaginal, Natural orifice specimen extraction (NOSE), Laparoscopic surgery, Cosmesis

## Abstract

**Background:**

This study aimed to evaluate cosmetic outcomes and feasibility of transvaginal natural orifice specimen extraction (NOSE) in patients who underwent laparoscopic or robotic surgery for the treatment of benign or malignant diseases of the kidney, liver, stomach, adrenal gland, and bladder.

**Methods:**

This prospective study was conducted at a tertiary hospital between March 2015 and May 2020. The main outcome was cosmetic outcomes of scars assessed using the Patient and Observer Scar Assessment Scale (POSAS) 1 and 8 weeks after surgery. The secondary outcomes were postoperative pain, operating time, and complications. Sexual function was assessed using the Female Sexual Function Index (FSFI) questionnaire 6 months after surgery in 17 patients who were sexually active at the time of surgery.

**Results:**

A total of 38 transvaginal NOSE procedures were performed for the extraction of 33 kidneys, 2 livers, 1 stomach, 1 adrenal gland, and 1 bladder. Observers rated pigmentation and relief scores as most deviant from normal skin (2.9 ± 1.7, 3.0 ± 2.1 at postoperative 1 week; 3.6 ± 1.9, 3.5 ± 2.2 at postoperative 8 weeks, respectively), but the overall scores of each item were low. The patients’ overall satisfaction with postoperative scars was high, and the mean scores for pain and itching were low, with significant improvement from the first week to the eighth week (*P* = 0.014 and *P* = 0.006, respectively). Patients also reported low scores on vaginal assessment items, indicating better symptoms, and bleeding improved significantly between the two time points (P = 0.001). Postoperative pain was reduced from moderate during the first 24 h after surgery to mild after 24 h. The mean operative time of the transvaginal NOSE procedure was 28.3 ± 13.3 min. No postoperative complications were associated with the procedure. The mean FSFI total score was 21.2 ± 8.7 (cutoff score for dysfunction is 21), with higher scores indicating better sexual functioning.

**Conclusion:**

Transvaginal NOSE seems to be a feasible procedure with promising cosmetic benefits, for patients who undergo minimally invasive surgery for large organs including the kidney, liver, stomach, adrenal gland, and bladder. A prospective randomized clinical trial is needed to provide solid evidence to support transvaginal NOSE.

**Trial registration:**

This trial is registered at ClinicalTrials.gov (NCT05113134).

## Background

Minimally invasive surgeries have been performed for benign and malignant diseases to minimize complications, shorten recovery times, and decrease scars after surgery [1]. When performing multiport laparoscopic surgery for large organs such as the stomach, colon, kidney, liver, and spleen, the extraction of surgical specimens requires an additional or enlarged abdominal wall incision to remove the resected organ or tissue. Enlargement of the incision may cause abdominal pain, infection, and incisional hernia during the postoperative period [2]. Natural orifice specimen extraction (NOSE) has been suggested to reduce incision-related morbidity and maximize the advantages of laparoscopic surgery [1].

Among various developed NOSE techniques, gynecologists have most widely selected the transvaginal access route through a posterior colpotomy incision [2, 3]. The posterior vaginal fornix is a large recess behind the cervix, a relatively accessible part of the vagina, and has good healing ability due to adequate vascular supply [4]. Previous studies have demonstrated the potential advantages of the transvaginal NOSE such as reduced postoperative pain, surgical site infection, improved patient recovery, better cosmetic results and lower incisional hernia rates [2]. However, concerns remain regarding sexual dysfunction and postoperative complications associated with colpotomy incision [4]. The use of this technique in minimally invasive surgery for large specimens has not been extensively investigated to date, and further research is needed.

This study aimed to evaluate the cosmetic outcomes, postoperative complications, and sexual function after transvaginal NOSE in patients who underwent multiport laparoscopic or robotic surgery for resection of large organs, including the kidney, liver, stomach, adrenal gland, and bladder.

## Methods

### Study design and setting

This prospective, single-center, cohort study was conducted at Seoul National University Bundang Hospital, a tertiary hospital in Korea, between March 2015 and May 2020. The study was approved by the Institutional Review Board of Seoul National University Bundang Hospital (No. B-1411-276-005) and was performed in accordance with the principles of the Declaration of Helsinki. All patients were adequately informed of the benefits and risks of the procedure and written consent was obtained prior to the surgical procedure.

### Study population

The inclusion criteria were as follow: patients over 20 years of age; patients with a distensible vagina that would permit the extraction of the surgical specimen; patients scheduled for laparoscopic resection of the stomach, liver, adrenal gland, bladder, colon, kidney, and spleen for benign or malignant diseases; and patients with normal cervical cancer screening tests (except inflammatory findings) within the last 3 years. The exclusion criteria were as follow: patients that have not had sexual intercourse throughout their life; patients with a narrow introitus noted on gynecological examination which would prevent removal of the specimen through the vagina; patients who were expected to have severe adhesions due to deep infiltrative endometriosis or previous pelvic surgery history; patients with abnormal cervical cancer screening tests; and patients scheduled to undergo concomitant hysterectomy.

### Operative technique

Under general anesthesia, multiport (robot-assisted) laparoscopic surgery was performed and the resected specimen was placed in an endopouch by specialized surgeons. Following the resection procedure, transvaginal NOSE is performed by gynecologic team. For the NOSE procedure, patients are placed in the Trendelenburg and lithotomy position. An approximately 1–2 cm incision was made at the posterior vaginal fornix through vaginal approach (posterior colpotomy), and laparoscopic forceps were introduced into the abdominal cavity through the colpotomy site. The thread of endopouch in the abdominal cavity was grasped by the laparoscopic forceps and was taken down out of the body cavity through the vagina. While pulling the thread down, the initial incision at the posterior vaginal fornix was laterally extended enough for the specimen extraction using fingers. The specimen inside the endopouch was removed through the vagina in the same way as the normal vaginal delivery of baby. Colpotomy closure was achieved transvaginally with a 2/0 absorbable suture. For this study, all procedures were performed by a single gynecologic surgeon.

### Data collection

We collected clinicopathological data from the patients’ medical records, such as age, body mass index (BMI), surgical history, history of keloid scars, perioperative outcomes including estimated blood loss, requirement of transfusion and hemoglobin level, pathologic reports, and treatment outcomes.

The primary endpoint was cosmetic outcomes of scars assessed using the Patient and Observer Scar Assessment Scale (POSAS) 1 and 8 weeks after surgery. POSAS is an internationally validated scar assessment questionnaire that measures the quality of a scar from the perspective of both patients and observers [5]. The POSAS consists of two distinct scales: OSAS (Observer Scar Assessment Scale) and PSAS (Patient Scar Assessment Scale). The OSAS includes five variables: vascularity, pigmentation, thickness, relief, and pliability. The PSAS includes six variables: scar-related pain, itching, color, stiffness, thickness, and irregularity. Each scar characteristic has a 10-point scoring system ranging from the lowest score of 1, representing normal skin, to the highest score of 10, representing the largest difference from normal skin. The total score of both scales is calculated by summing the items, ranging from 5 to 50 for the OSAS and 6 to 60 for the PSAS. In addition, patients ranked their overall opinion of the scars ranging from 1 to 10, with 1 representing the best scar imaginable and 10 the worst scar imaginable. These were not included in the total score. For vaginal wound evaluation, we created a questionnaire consisting of three items: vaginal bleeding, discharge, and pain. Each item was graded on a 3-point scale ranging from 1 (minimal symptoms) to 3 (maximum symptoms). The total score represented the addition of scores for all items ranging from three to nine.

The secondary outcomes were total duration of operating time, operating time for transvaginal NOSE, postoperative pain, postoperative complications, and analgesic needs 2, 6, 24, and 48 h after surgery. Six months after surgery, sexual function was assessed using the Female Sexual Function Index (FSFI) questionnaire. The FSFI consists of 19 items that measure female sexual function in six domains: desire, arousal, lubrication, orgasm, satisfaction, and pain [6]. The total score is the sum of six domains, with a maximum score of 36. The lower scores represent worse sexual function. A total score of 21 has been validated as the cutoff score for the diagnosis of female sexual dysfunction [6].

### Statistical analysis

The normality of the distribution was determined using the Kolmogorov-Smirnov test. Student’s *t*-test and Mann-Whitney *U* test were used to compare continuous parametric and non-parametric variables, respectively. Pearson’s chi-square test or Fisher’s exact test was used to compare categorical variables. All analyses were performed using the SPSS software for Windows (version 25.0; SPSS Inc., Chicago, IL, USA). Statistical significance was set at *p* < 0.05.

## Results

**Fig. 1:**
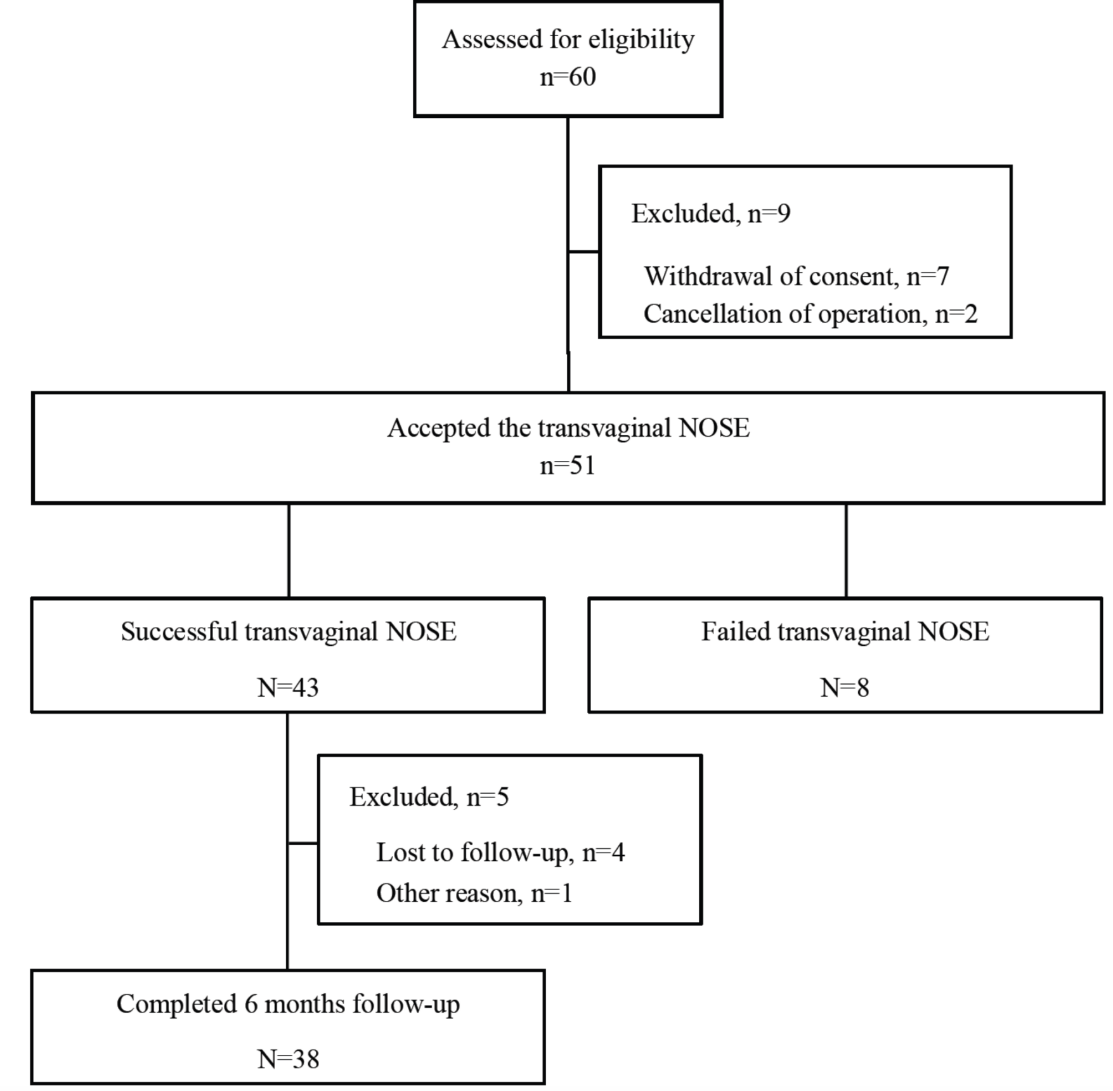
Study flow chart. NOSE, natural orifice specimen extraction

Sixty patients were assessed for eligibility, and 9 patients were excluded due to withdrawal of consent and cancellation of the operation (Fig. 1). Among 51 patients, 8 (15.7%) patients failed to undergo the transvaginal NOSE procedure in unexpected circumstances, for example, obliteration of posterior cul-de-sac and relatively narrow vaginal cavity compared with bulky specimen which was not pre-planned at the time of gynecologic examination. Of the 43 enrolled patients, five withdrew from the study because of loss to follow-up (3 patients lost to contact, 1 patient self-transferred to another clinic) or prolonged postoperative intensive care unit stay. Finally, 38 patients completed the 6-months follow up.

Baseline demographics and characteristics are shown in Table 1. The mean (± standard deviation [SD]) age and body mass index (BMI) were 57.6 ± 14.1 years and 23.5 ± 2.7 kg/m^2^, respectively. Thirteen (34.2%) and 1 (2.6%) patient had a history of abdominal surgery and keloids, respectively. The transvaginal NOSE procedure was performed to extract 33 kidneys, 2 livers, 1 stomach, 1 adrenal gland, and 1 bladder tissue. Histopathology was identified as benign in 8 (21.1%) and malignant in 30 (78.9%) patients. Of the 30 patients with malignant disease, 3 (10.0%) experienced recurrence other than at the vaginal site during a median length of observation of 32.3 months (range, 7.2–73.1 months).

**Table 1:** Characteristics of overall patients (n = 38)

Characteristics	Value
Age, years	57.6 ± 14.1
BMI, kg/m^2^	23.5 ± 2.7
Previous abdominal surgery	13 (34.2)
Previous keloid history	1 (2.6)
Extracted specimen	
Kidney	33 (86.8)
Radical nephrectomy	8 (21.1)
Simple nephrectomy	11 (28.9)
Partial nephrectomy	14 (36.8)
Liver	2 (5.3)
Hemihepatectomy	1 (2.6)
Sectionectomy	1(2.6)
Stomach	1 (2.6)
Distal gastrectomy	1 (2.6)
Adrenal gland	1 (2.6)
Adrenalectomy	1 (2.6)
Bladder	1 (2.6)
Partial cystectomy	1 (2.6)
Histopathology	
Benign	8 (21.1)
Malignant	30 (78.9)
Recurrence in Malignant disease	
Yes	3 (10.0)
No	27 (10.0)

BMI, body mass index

Values are presented as mean ± standard deviation or n (%) unless otherwise indicated.

The surgical outcomes are described in Table 2. The mean (± SD) total operation duration and operative time for the transvaginal NOSE procedure were 233.8 ± 78.2 min and 28.3 ± 13.3 min, respectively. The mean (± SD) estimated blood loss was 163.7 ± 162.0 mL, and blood transfusions were required in 4 (10.5%) and 8 (21.1%) patients intraoperatively and postoperatively, respectively. The mean drop of hemoglobin was 1.1 ± 1.4 g/dL before and 24 h after operation. The median number of trocar ports was 5 (range, 2–5) with a mean (± SD) incision length of 1.4 ± 0.3 cm. Although the cumulative PCA dose continued to increase postoperatively from 2 to 48 h, the proportion of patients who had asked for extra-painkiller showed plateau around 25% throughout the monitoring period. The Visual Analog Scale (VAS) score gradually improved from moderate to mild pain. No intraoperative complications occurred; however, 2 patients had early postoperative complications and 1 patient had late postoperative complications which were not associated with the transvaginal NOSE procedure. The mean (± SD) gas passing and hospital stay days were 2.2 ± 1.0 days and 6.7 ± 1.7 days, respectively.

**Table 2:** Surgical outcomes

Variable	Value
Operating time, min	233.8 ± 78.2
Transvaginal NOSE time, min	28.3 ± 13.3
Estimated blood loss, mL	163.7 ± 162.0
Transfusion	
Intraoperative	4 (10.5)
Postoperative	8 (21.1)
Hemoglobin level, g/dL	
Preoperative	12.1 ± 1.6
Postoperative	10.9 ± 1.3
Preoperative-postoperative	1.1 ± 1.4
Scar	
Port number, median (range)	5 (2–5)
Incision length, cm	1.4 ± 0.3
2 h postoperative period	
VAS	5.3 ± 2.1
PCA amount, mL	6.9 ± 6.7
Painkiller consumption	9 (23.7)
6 h postoperative period	
VAS	4.9 ± 1.8
PCA amount, mL	11.5 ± 4.3
Painkiller consumption	8 (21.1)
24 h postoperative period	
VAS	3.5 ± 1.8
PCA amount, mL	29.4 ± 12.1
Painkiller consumption	12 (31.6)
48 h postoperative period	
VAS	2.5 ± 1.5
PCA amount, mL	52.3 ± 19.3
Painkiller consumption	9 (23.7)
Postoperative complication	3 (7.9)
Acute kidney injury	1 (2.6)
Delirium	1 (2.6)
Epidermal cyst	1 (2.6)
Postoperative recovery	
Gas passing day	2.2 ± 1.0
Hospital stay, day	6.7 ± 1.7

NOSE, natural orifice specimen extraction; VAS, visual analog scale; PCA, patient-controlled analgesia

Values are presented as mean ± standard deviation or n (%) unless otherwise indicated.

**Fig. 2:**
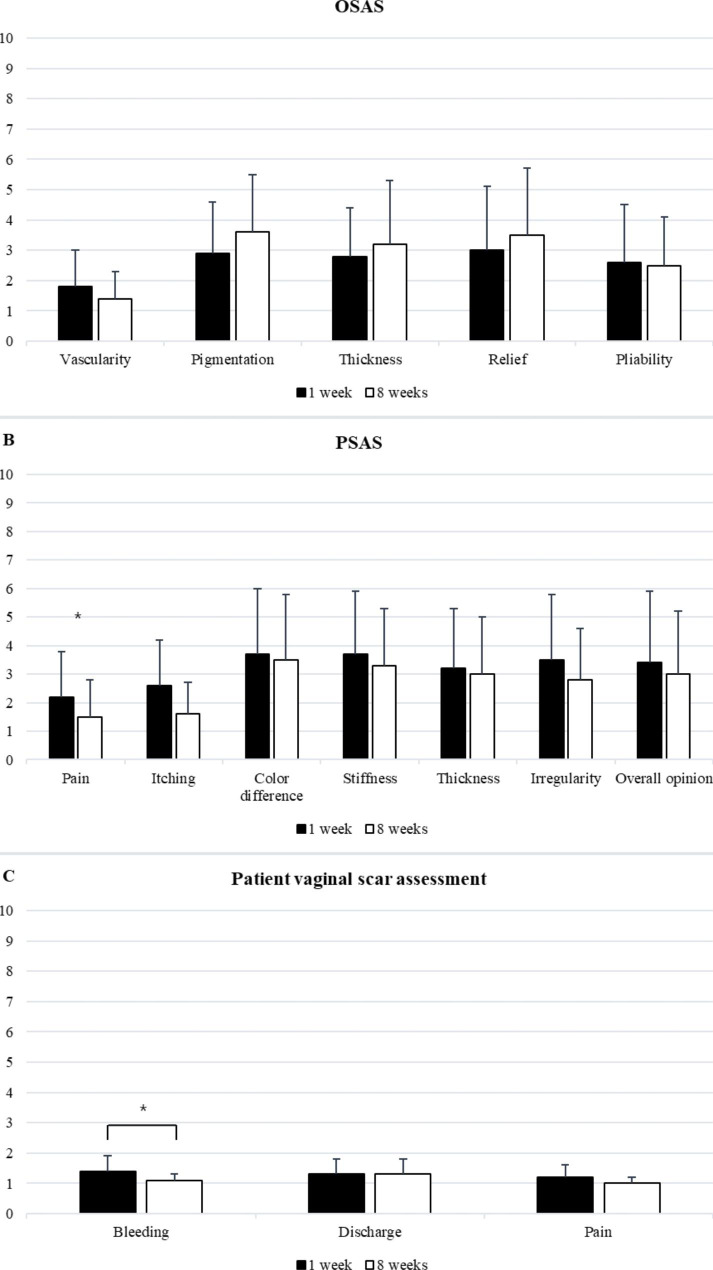
Scar assessments. (**A**) Observer scar assessment scale at 1 week and 8 weeks after surgery (mean ± standard deviation). (**B**) Patient scar assessment scale at 1 week and 8 weeks after surgery (mean ± standard deviation). (**C**) Patient vaginal scar assessment at 1 week and 8 weeks after surgery (mean ± standard deviation). OSAS, Observer Scar Assessment Scale; PSAS, Patient Scar Assessment Scale

Observers rated the scores of pigmentation and relief as most deviant from normal skin (2.9 ± 1.7, 3.0 ± 2.1 at postoperative 1 week; 3.6 ± 1.9, 3.5 ± 2.2 at postoperative 8 weeks, respectively), but the overall scores of each items were low (Table 3; Fig. 2 A). The patients’ overall satisfaction regarding postoperative scars was high (3.4 ± 2.5 at postoperative 1 week; 3.2 ± 2.2 at postoperative 8 weeks) and the mean scores of pain and itching were low with improvement between 1 week and 8 weeks (*P* = 0.014, *P* = 0.006, respectively) (Table 3; Fig. 2B). Patients also reported low scores for vaginal assessment items, and bleeding improved significantly between the two time points (*P* = 0.001) (Table 3; Fig. 2 C). Seventeen patients who underwent vaginal NOSE were sexually active at the time of the surgery. The mean FSFI total score was 21.2 ± 8.7, above the cutoff for sexual dysfunction (defined as 21) (Table 4).

**Table 3:** Postoperative scar assessments

Variables	Postoperative 1 week	Postoperative 8 weeks	*P* value
OSAS^*^			
Vascularity	1.8 ± 1.2	1.4 ± 0.9	0.082^b^
Pigmentation	2.9 ± 1.7	3.6 ± 1.9	0.048^b^
Thickness	2.8 ± 1.6	3.2 ± 2.1	0.635^b^
Relief	3.0 ± 2.1	3.5 ± 2.2	0.251^b^
Pliability	2.6 ± 1.9	2.5 ± 1.6	0.558^b^
Total score	13.0 ± 7.1	14.1 ± 7.4	0.825^b^
PSAS^*^			
Pain	2.2 ± 1.6	1.5 ± 1.3	0.014^b^
Itching	2.6 ± 1.6	1.6 ± 1.1	0.006^b^
Color difference	3.7 ± 2.3	3.5 ± 2.3	0.767^a^
Stiffness	3.7 ± 2.2	3.3 ± 2.0	0.525^a^
Thickness	3.2 ± 2.1	3.0 ± 2.0	0.766^a^
Irregularity	3.5 ± 2.3	2.8 ± 1.8	0.057^a^
Total score	18.9 ± 9.3	15.7 ± 8.2	0.053^a^
Overall opinion	3.4 ± 2.5	3.0 ± 2.2	0.314^a^
Vaginal complications^†^			
Bleeding	1.4 ± 0.5	1.1 ± 0.2	0.001^b^
Discharge	1.3 ± 0.5	1.3 ± 0.5	> 0.999 ^b^
Pain	1.2 ± 0.4	1.0 ± 0.2	0.096 ^b^
Total score	3.9 ± 1.1	3.4 ± 0.6	0.006^b^

OSAS, observer scar assessment scale; VAS, visual analog scale; PSAS, patient scar assessment scale; PVSAS, patient vaginal scar assessment scale

Values are presented as mean ± standard deviation.

^a^ P values were calculated by paired t-test.

^b^ P values were calculated by Wilcoxon signed rank test.

^*^ Each scar characteristics of scar assessment scale has a 10-point scoring system ranges from lowest score 1 representing the situation of normal skin to highest score 10 representing largest difference from normal skin.

^†^ Each items graded on a 3-point scale ranging from 1 indicating minimal symptoms to 3 indicating maximum symptoms.

**Table 4:** Postoperative sexual function assessments

Variables (score range)	Value
Sexually active women	17 (44.7)
FSFI^*^	
Desire (1.2-6)	2.6 ± 0.9
Arousal (0–6)	4.1 ± 1.7
Lubrication (0–6)	3.4 ± 1.8
Orgasm (0–6)	3.6 ± 1.9
Satisfaction (0.8-6)	4.5 ± 1.8
Pain (0–6)	3.0 ± 1.8
Total score (2–36)	21.2 ± 8.7

FSFI, female sexual function index

Values are presented as mean ± standard deviation or n (%) unless otherwise indicated

^*^ FSFI consist of 19 items that measures female sexual function in 6 domains. The total score is a maximum score of 36 with lower scores representing poor sexual function

## Discussion

Our study showed that transvaginal NOSE is a feasible procedure with promising cosmetic benefits for extracting large organ specimens for both benign and malignant diseases in patients undergoing multiport laparoscopic or robotic surgery. Compared to open surgery, the laparoscopic approach generally results in less postoperative pain, shorter recovery time, and improved cosmetic outcomes [7]. However, multiport laparoscopic surgery often requires an additional or enlarged abdominal wall incision for specimen extraction. Enlargement of the abdominal wall incision can lead to postoperative complications, including postoperative pain, surgical site infections, incisional hernias, and cosmetic problems [2, 3]. NOSE can address these problems when surgeons incorporate it into existing minimally invasive surgical procedures.

Previous studies have shown that NOSE results in faster recovery, shorter hospital stay, better postoperative pain control, fewer incisional complications, and improved cosmesis [4]. In addition, NOSE can resolve the difficulty in transabdominal specimen removal in patients with deep abdominal walls [8]. Although the feasibility of NOSE has been demonstrated, some potential concerns remain.

One of the main concerns when extracting malignant disease specimens via transvaginal NOSE is the fear of implantation of tumor cells within the vaginal extraction site. Consistent with our results, other studies have reported that NOSE is safe for malignant diseases. A systematic review concluded that there was no significant difference in oncologic outcomes between NOSE and abdominal incision groups [9]. One study reported similar overall survival and disease-free survival between the two approaches, and another study reported no tumor implantation problems during the follow-up period after the NOSE procedure [10, 11]. The use of a protective bag before specimen extraction would reduce the risk of cancer cell implantation.

Another major concern of transvaginal NOSE is the possibility of postoperative sexual dysfunction. Previous studies evaluating transvaginal NOSE in laparoscopic nephrectomy and nephroureterectomy reported there were no significant changes in sexual function [12, 13]. Our study showed low FSFI scores, however, still higher than the cutoff of 21 for sexual dysfunction. We did not measure the preoperative FSFI score; therefore, it is difficult to determine whether the procedure lowered the score from baseline. In a study conducted on patients who underwent transvaginal NOSE in laparoscopic partial or radical nephrectomy, the mean preoperative and postoperative FSFI scores were 21.6 and 21.8 respectively, which were similar to the scores in our study [11]. As innervation in the posterior vaginal fornix is sparse, transvaginal NOSE may not affect sexual impulses or cause dyspareunia.

Consistent with our results, most previous studies have reported that postoperative infections were not related to posterior colpotomy and transvaginal extraction [14–16]. Conversely, the complication of vaginal abscess has been reported after transvaginal NOSE procedure following laparoscopic myomectomy [17, 18]. However, the authors explained that the abscess could be attributed to the adhesion barrier used during surgery. Theoretically, increased exposure time of the abdominal cavity to vaginal cavity during NOSE procedure might cause possible contamination by vaginal microorganisms. However, this concern could be dismissed because the positive intraabdominal pressure generated by pneumoperitoneum may prevent peritoneal bacterial contamination [2].

Vagina is one of the best routes for surgical specimen extraction. However, it is important to properly select good candidates for successful transvaginal NOSE. In the current study, all patients who met the eligibility criteria and signed the informed consent received gynecologic evaluation for the adequacy of transvaginal NOSE. Vaginal examination using a speculum and transvaginal ultrasound had been performed by the gynecologic surgeon who was responsible for transvaginal NOSE. A negative result of Papanicolau test within the last 1 year was confirmed for all of the patients. The sizes of tumor on preoperative CT scan and vaginal cavity were considered together with the surgical plan in order to assess the success rate of transvaginal NOSE. In this study, transvaginal NOSE procedure was abandoned in 15.7% (8/51) because of unexpected intraoperative situations: (1) failure of posterior culdotomy because of inaccessible posterior cul-de-sac; (2) severe pelvic adhesion without previous operation history; and (3) relatively narrow vaginal cavity compared with bulky specimen by radical excision together with surrounding tissue which was not pre-planned at the time of gynecologic examination. Success of transvaginal NOSE reportedly depends on various patient factors such as sex, BMI, medical comorbidities and prior medical history. Patients with high BMI are associated with increased visceral fat, which is related to bulky specimen and may lead to failure of this procedure [19]. Patients with more comorbidities are vulnerable to complications and history of prior surgeries and radiation exposure may influence the feasibility of transvaginal NOSE [19]. Specimen factors including whole specimen diameter and shape may also determine the success of transvaginal NOSE procedure. Previous studies report greater success rate of the transvaginal NOSE with a diameter smaller than 9 cm [19, 20].

Our study has some limitations. First, transvaginal NOSE was performed by a single surgeon with advanced experience in vaginal surgery. Therefore, experience in vaginal surgery is required to optimize results. Second, to minimize dropouts due to the burden of completing the questionnaires, we did not perform a preoperative FSFI questionnaire. The lack of information from the questionnaire prevented us draw valid conclusions about sexual function in our patients apart from the fact that the scores obtained are low. Finally, the specimen size, which we did not measure, may be a restriction in applying the transvaginal NOSE procedure. However, in other studies, specimen mass size was not an important determinant of the success of NOSE [21, 22].

## Conclusion

Due to the widespread advancement of laparoscopic and robotic surgery in recent years, most surgeons have experience in minimally invasive surgery, which has led to the development of NOSE procedures. In performing transvaginal NOSE, we found that the procedure resulted in good cosmetic outcomes and seemed feasible with favorable surgical outcomes, even in cases with malignant diseases, when combined with minimal invasive surgery that required extraction of large organ specimens.

## Data Availability

The datasets used and/or analyzed during the current study are available from the corresponding author on reasonable request.

## Abbreviations

NOSE: natural orifice specimen extraction.
POSAS: Patient and Observer Scar Assessment Scale.
OSAS: Observer Scar Assessment Scale.
PSAS: Patient Scar Assessment Scale.
FSFI: Female Sexual Function Index.
BMI: body mass index.
VAS: visual analog scale.
SD: standard deviation.
